# Digital Volume Pulse Measured at the Fingertip as an Indicator of Diabetic Peripheral Neuropathy in the Aged and Diabetic

**DOI:** 10.3390/e21121229

**Published:** 2019-12-16

**Authors:** Hai-Cheng Wei, Na Ta, Wen-Rui Hu, Ming-Xia Xiao, Xiao-Jing Tang, Bagus Haryadi, Juin J. Liou, Hsien-Tsai Wu

**Affiliations:** 1 School of Electrical and Information Engineering, North Minzu University, No. 204 North Wenchang Street, Yinchuan, Ningxia 750021, China; wei_hc@nun.edu.cn (H.-C.W.); ta_na@nmu.edu.cn (N.T.); 20187144@stu.nun.edu.cn (W.-R.H.); xiao_mx@nmu.edu.cn (M.-X.X.); jjliou07@gmail.com (J.J.L.); 2 Basic Experimental Teaching and Engineering Training Center, North Minzu University, No. 204 North Wenchang Street, Yinchuan, Ningxia 750021, China; 3 School of Science, Ningxia Medical University, No. 1160 Shengli Street, Ningxia 750004, China; tangxj@nxmu.edu.cn; 4 Department of Physics, Universitas Ahmad Dahlan, Jendral A. Yani street, Kragilan, Tamanan, Kec. Banguntapan, Bantul, Daerah Istimewa, Yogyakarta 55191, Indonesia; bagus.haryadi@fisika.uad.ac.id; 5 College of Electronics and Information Engineering, Shenzhen University, Shenzhen 518060, China; 6 Department of Electrical Engineering, Dong Hwa University, No. 1, Sec. 2, Da Hsueh Rd., Shoufeng, Hualien 97401, Taiwan, China

**Keywords:** percussion entropy index, baroreflex sensitivity, autonomic nervous function, digital volume pulse, photoplethysmography, peak-to-peak interval, ensemble empirical mode decomposition, diabetic peripheral neuropathy

## Abstract

This study investigated the application of a modified percussion entropy index (PEI_PPI_) in assessing the complexity of baroreflex sensitivity (BRS) for diabetic peripheral neuropathy prognosis. The index was acquired by comparing the obedience of the fluctuation tendency in the change between the amplitudes of continuous digital volume pulse (DVP) and variations in the peak-to-peak interval (PPI) from a decomposed intrinsic mode function (i.e., IMF6) through ensemble empirical mode decomposition (EEMD). In total, 100 middle-aged subjects were split into 3 groups: healthy subjects (group 1, 48–89 years, n = 34), subjects with type 2 diabetes without peripheral neuropathy within 5 years (group 2, 42–86 years, n = 42, HbA1c ≥ 6.5%), and type 2 diabetic patients with peripheral neuropathy within 5 years (group 3, 37–75 years, n = 24). The results were also found to be very successful at discriminating between PEI_PPI_ values among the three groups (*p* < 0.017), and indicated significant associations with the anthropometric (i.e., body weight and waist circumference) and serum biochemical (i.e., triglycerides, glycated hemoglobin, and fasting blood glucose) parameters in all subjects (*p* < 0.05). The present study, which utilized the DVP signals of aged, overweight subjects and diabetic patients, successfully determined the PPI intervals from IMF6 through EEMD. The PEI_PPI_ can provide a prognosis of peripheral neuropathy from diabetic patients within 5 years after photoplethysmography (PPG) measurement.

## 1. Introduction

Microvascular diseases are prevalent among patients with long-term type 2 diabetes mellitus [1,2]. In general, diabetic microvascular diseases are classically characterized by exudate leakage from retinal small vessels (i.e., diabetic retinopathy), persistent proteinuria and progressive decline in kidney function (i.e., diabetic nephropathy), and diabetic peripheral neuropathy (DPN). DPN is one of the most common chronic complications of diabetes [3,4]. Not only is the quality of life much lower among type 2 diabetes mellitus patients with cardiovascular complications and DPN, but the mortality rate has also been shown to be higher than either illness alone [5]. Early detection of the signs of DPN through signal analysis methods, therefore, is of utmost urgency. Moreover, type 2 diabetic patients were reported to be at increased risk of developing atherosclerosis and autonomic nervous dysfunction [6,7,8,9]. Hence, those parameters addressed for atherosclerosis and autonomic nervous dysfunction measurements were considered for DPN prediction in the study.

**Figure 1:**
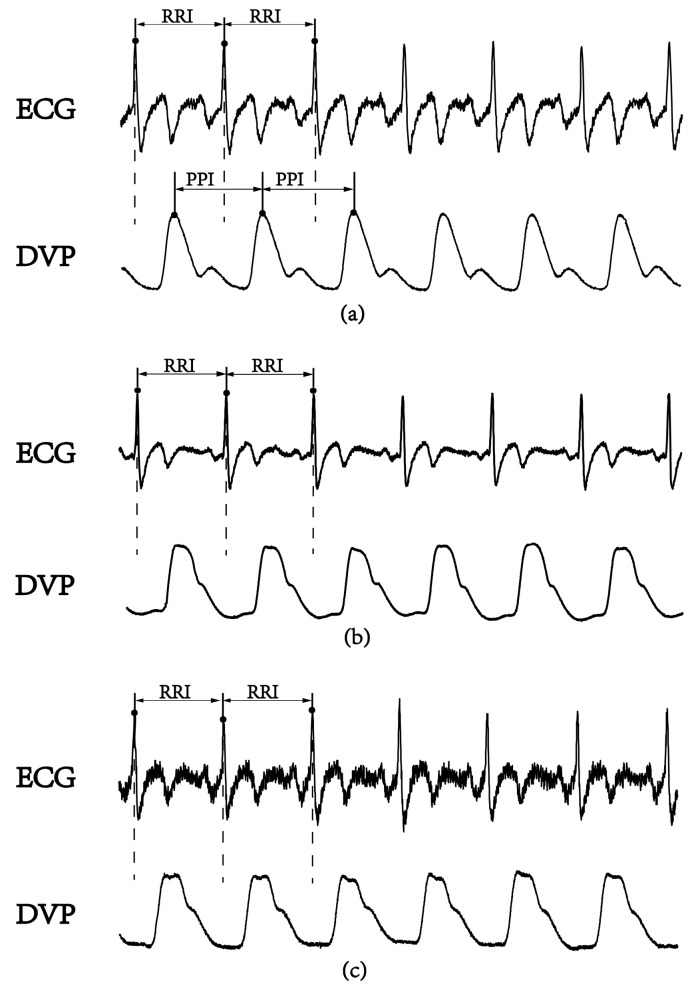
A lead II electrocardiogram (ECG) obtained using the conventional method and synchronous volume pulse from an infrared photoplethysmography (PPG) sensor attached to the dominant index finger. The PPG-derived digital volume pulse (DVP) signals for certain time periods are shown. R-R interval: the period between consecutive ECG R waves; peak-to-peak interval (PPI): the period between the peaks of two consecutive volume pulses. (**a**) Healthy subject (age: 52 years), (**b**) overweight and elderly subject (age: 66 years), and (**c**) type 2 diabetic patient (age: 42 years).

In the frequency domain analysis of heart rate variability (HRV), R-R interval (RRI) measurement is used as a conventional method of autonomic function and baroreflex sensitivity (BRS) assessments [10]. The low-/high-frequency power ratio (LFP/HFP, LHR) is the index for frequency domain analysis. The LHR index is considered to reflect human autonomic failure [10,11], because physiological signals from the human body are almost nonstationarity and nonlinear [12,13,14]. Recently, several new parameters based on nonlinear HRV calculations were reported for autonomic function and BRS assessments [15,16,17]. Among these parameters, a small-scale multiscale entropy index (MEI_RRI_) was addressed to reflect on autonomic function based on a nonlinear method for studying HRV using only the RRI datasets [15]. In recent years, the percussion entropy index (PEI) using synchronized ECG and photoplethysmography (PPG) signals has been used effectively to assess BRS complexity in the aged and diabetic patients associated with type 2 diabetes-associated autonomic dysfunction [16,17]. The peak-to-peak intervals (PPIs) acquired by the PPG sensor were reported to assess HRV [18,19,20,21,22,23]. Moreover, PPG-derived digital volume pulse (DVP) signals were further used for clinical applications (i.e., ubiquitous blood pressure monitoring, congestive heart failure, and hypertension assessment) [24,25,26]. However, the compatibility of PPI with RRI is controversial [18,23]. In general, PPI could provide surrogate data for RRI as an alternative means of evaluating cardiac autonomic function in healthy young individuals with normal BMIs. However, it appears to be inappropriate to use PPI to replace RRI for overweight, elderly, and diabetic individuals [18,19]. In Figure 1, we can observe the failure of the digital volume pulse to detect the peak-to-peak interval (PPI) for an aged, overweight subject and a diabetic subject, as reflected in some of its subtle corresponding peaks; this means a further process is needed for PPI to be a surrogate of RRI.

Although the above algorithms have been reported to increase the accuracy of PPI, most efforts have been made to reduce computation load [21,22,23]. The objectives of this study are to test two hypotheses. First, we hypothesize that the ensemble empirical mode decomposition (EEMD) method could be utilized for PPG-derived DVP signals to obtain an intrinsic mode function with cardiac cycle information in overweight, elderly, and diabetic individuals. Using synchronized RRI and PPG-derived amplitude series, a previous study successfully identified poor blood-glucose-controlled diabetic patients with a significantly low percussion entropy index (i.e., PEI_RRI_) [16]. Our second hypothesis is that a modified percussion entropy index (i.e., PEI_PPI_) in which PPG-derived PPI series and PPG-derived amplitude series were adopted could provide a prognosis of diabetic peripheral neuropathy for diabetic patients as much as 5 years in advance.

This paper describes the surrogate data for percussion entropy in assessing the complexity of BRS for diabetic peripheral neuropathy prognosis. Descriptions of the study population, study protocol, the modified percussion entropy index using synchronized {PPI} and {Amp} signals, and statistical analysis are presented in Section 2. Baseline characteristics of the age-controlled healthy and diabetic subjects; failure of DVPs in detecting peak-to-peak intervals in an aged overweight subject and a diabetic subject; agreement assessment between PPI (DVP) and PPI (IMF6) for the three groups; and comparisons among PEI_PPI_, PEI_RRI_, MEI_RRI_, and LHR_RRI_ to differentiate future peripheral neuropathy from type 2 diabetic patients are addressed in Section 3. Our findings are discussed in Section 4. Section 5 concludes the manuscript. 

## 2. Materials and Methods 

### 2.1. Study Population

In total, 108 right-hand dominant, middle-aged subjects were prospectively recruited for ECG and PPG examinations at Hualien Hospital (Hualien City, Taiwan) between June 2009 and June 2011. Of the 108 subjects recruited, 8 participants were excluded due to a history of atherosclerosis-associated complications, including ischemic stroke, coronary heart disease, peripheral vascular disease, atrial fibrillation, heart failure, and permanent pacemaker implantation, with 100 subjects remaining for the study. Diabetes mellitus was defined as a fasting glucose level higher than 126 mg/dL and an HbA1c level larger than 6.5%. Of these, 34 middle-aged subjects were not diabetic patients (group 1, age range: 48–89 years, n = 34), and 66 had type 2 diabetes. The diabetic patients were then divided into two groups; namely, middle-aged subjects diagnosed as having type 2 diabetes without peripheral neuropathy within 5 years (group 2, age range: 42–86 years, n = 42, glycated hemoglobin (HbA1c) ≥ 6.5%), and type 2 diabetic patients with peripheral neuropathy within 5 years (group 3, age range: 37–75 years, n = 24). There were 10 diabetic patients in group 3 with good blood glucose control (i.e., 6.5% ≦ HbA1c < 8%) in our present study, and 14 diabetic patients who originally had poor blood glucose control (i.e., HbA1c ≧ 8%) [17]. The screening DPN of type 2 diabetes patients at baseline of PPG measurement and follow-up periods (i.e., 5 years) was based on the presence of symptoms of numbness, tingling, or pain of distal extremities lasting for more than 3 months, along with a confirmed diagnosis by the clinic doctor. For unbiased analysis, the subjects in the three groups were age-controlled. This study was reviewed and approved by the Institutional Review Board of Hualien Hospital (Hualien City, Taiwan) and Ningxia Medical University Hospitals (Yinchuan City, Ningxia, China) (No. 2018-229). Each patient signed an informed consent.

### 2.2. Study Protocol

Demographic, anthropometric, and laboratory data for the analysis as well as medical history were obtained at the clinic visit. Body mass index was calculated as body weight (kilograms)/height (meters)^2^. Each participant’s resting blood pressure was measured once with the left arm in a supine position by a oscillometric device (BP3AG1, Microlife, Taiwan) with an appropriately sized cuff. Total cholesterol, triglycerides, low-density lipoprotein cholesterol, and high-density lipoprotein cholesterol concentrations were obtained from blood samples after a 12-h fast. Caffeine-containing beverages and theophylline-containing drugs were forbidden for 12 h before each hospital visit. All measurements were taken in the morning (i.e., 08:30–10:30). Moreover, to minimize latent erroneous readings from the PPG sensors arising from involuntary body vibrations of the test subjects and a low environmental temperature, possibly resulting in constriction of the peripheral vessels, all participants underwent blood sampling before data acquisition. All the test subjects were allowed to relax in a supine position for 5 min in a quiet room with the temperature controlled at 26 ± 1 °C. Data from the first 1000 cardiac cycles were used for analysis in the present study. 

### 2.3. Calculation of the Percussion Entropy Index (PEI_PPI_) Using Synchronized {PPI} and {Amp} Signals 

The PPG infrared sensor was applied to a dominant index fingertip of the subjects for data acquisition. After being performed through an USB-based analog-to-digital converter (USB-6009 DAQ, National Instruments, Austin, TX, USA) with a sampling frequency of 500 Hz, the digitized signals were subsequently stored on a personal computer (PC) and were analyzed by Matlab 7.7 software package (MathWorks, MA, USA) [27]. In each DVP cycle, the potential difference between the peak (the highest point determined by Matlab software) and the valley (the lowest point after the peak) was defined as the pulse amplitude of the DVP signals. The {PPI} series were calculated from IMF6 after decomposition of DVP signals by EEMD. The DVP and IMF6 signals had to be synchronized. Each peak and valley of the DVPs were obtained between two consecutive ECG R waves (Figure 1).

#### 2.3.1. Surrogate Data for Baroreflex: Synchronized {PPI} and {Amp} Signals

The baroreflex is one of the body’s homeostatic mechanisms that is reflected in a physiological phenomenon where a decrease in blood pressure makes the RRI shorter, and an increase in blood pressure makes the RRI longer. In our previous study [16,17], synchronized RRI and amplitude series from DVP signals were used to develop a percussion entropy index to assess diabetic autonomic nervous dysfunction. However, an ECG is not convenient for real-time application. In the current study, we attempted to generate a modified index using only DVP signals (i.e., PPI to replace RRI) under the concept of surrogate data and cost reduction.

**Figure 2:**
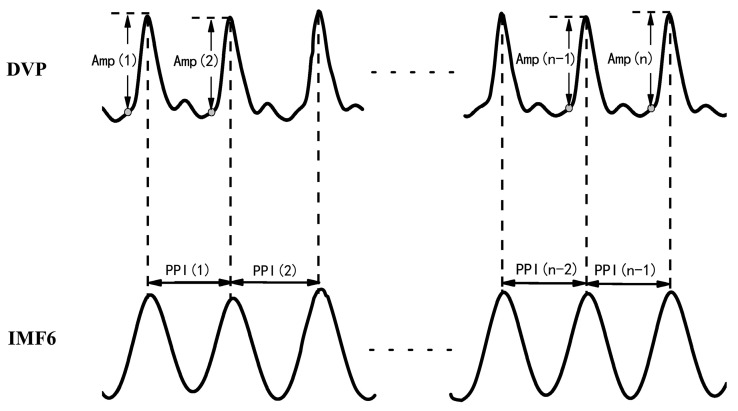
DVP amplitudes of the dominant finger {Amp(1), Amp(2), …, Amp(n)} and peak-to-peak intervals (PPIs) of IMF6 {PPI(1), PPI(2), …, PPI(n − 1)} were simultaneously acquired from a six-channel ECG-based pulse wave velocity system [28].

- Synchronized {PPI} and {Amp} signals
  {Amp} = {Amp(1), Amp(2),…, Amp(n)} for time series of DVP amplitude signals, and {PPI} = {PPI(1), PPI(2),…, PPI(n − 1)} for the PPI of IMF6 after EEMD, were simultaneously synchronized for each subject, as shown in Figure 2.
  {Amp} = {Amp(1), Amp(2), Amp(3), …, Amp(n)},(1)
  {PPI} = {PPI(1), PPI(2), PPI(3), …, PPI(n − 1)}.(2)

After EEMD was implemented via the Matlab package using a PC, the 6th intrinsic mode function (IMF6) was decomposed from DVPs, which are sine-wave-like, caused by the impact of heart pulsation on the DVP.

- Synchronized {BPPI} and {BAmp} signals for fluctuation t patterns
  Baroreflex sensitivity (BRS) had been shown to be a novel parameter of autonomic function. BRS can quantitatively reflect the matching degree between a change in DVP amplitudes of dominant fingers (i.e., {Amp} in Equation (1)) and a change in the peak-to-peak intervals (PPIs) of IMF6 (i.e., {PPI} in Equation (2)). Therefore, the fluctuations among successive DVP waveform amplitudes and PPIs from IMF6 undergo binary transformation to get two binary sequences (i.e., {B_Amp_} and {B_PPI_}, respectively). In that way, {B_Amp_} and {B_PPI_} represented the fluctuations of series {Amp} and {PPI}, respectively.

**Figure 3:**
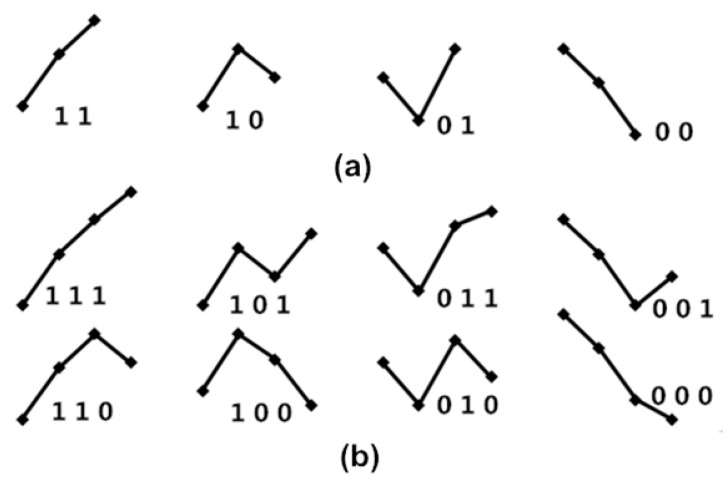
(**a**) The four fluctuation patterns of length two and (**b**) the eight fluctuation patterns of length three, representing fluctuations of {Amp} and {PPI} time series. Here, “1” represents Amp(i + 1) up from Amp(i); “0” represents Amp(i + 1) down from Amp(i) for the {Amp} series; “1” represents PPI(i + 1) increased from PPI(i); “0” represents PPI(i + 1) decreased from PPI(i) for the {PPI} series.

The four fluctuation patterns of length two, and the eight fluctuation patterns of length three, representing fluctuations of {Amp} and {PPI} time series, are shown in Figure 3. We used these patterns to calculate obedience in the fluctuation tendency for represented BRS.

{B_Amp_} = {a_1_ a_2_ a_3_ … a_n_}, a_i_ = 0, if Amp(I + 1) < Amp(i); or a_i_ = 1, if Amp(I + 1) > Amp(i), (3)

{B_PPI_} = {p_1_ p_2_ p_3_ ... p_n_}, p_i_ = 0, if PPI(i + 1) < PPI(i); or p_i_ = 1, if PPI(i + 1) > PPI(i). (4)

#### 2.3.2. Surrogate Data for Percussion Entropy in Assessing the Complexity of BRS

In a previous study [16], variations in BRS caused 1 to 5 cardiac cycle delays under the effects of blood pressure (i.e., DVP amplitude) changes on synchronized RRIs. Accordingly, obedience in the fluctuation tendency, the percussion entropy with a length of the fluctuation pattern equal to two, was represented as baroreflex sensitivity, while the percussion entropy with a length of the fluctuation pattern equal to three was represented as the complexity of a biological system.

**Figure 4:**
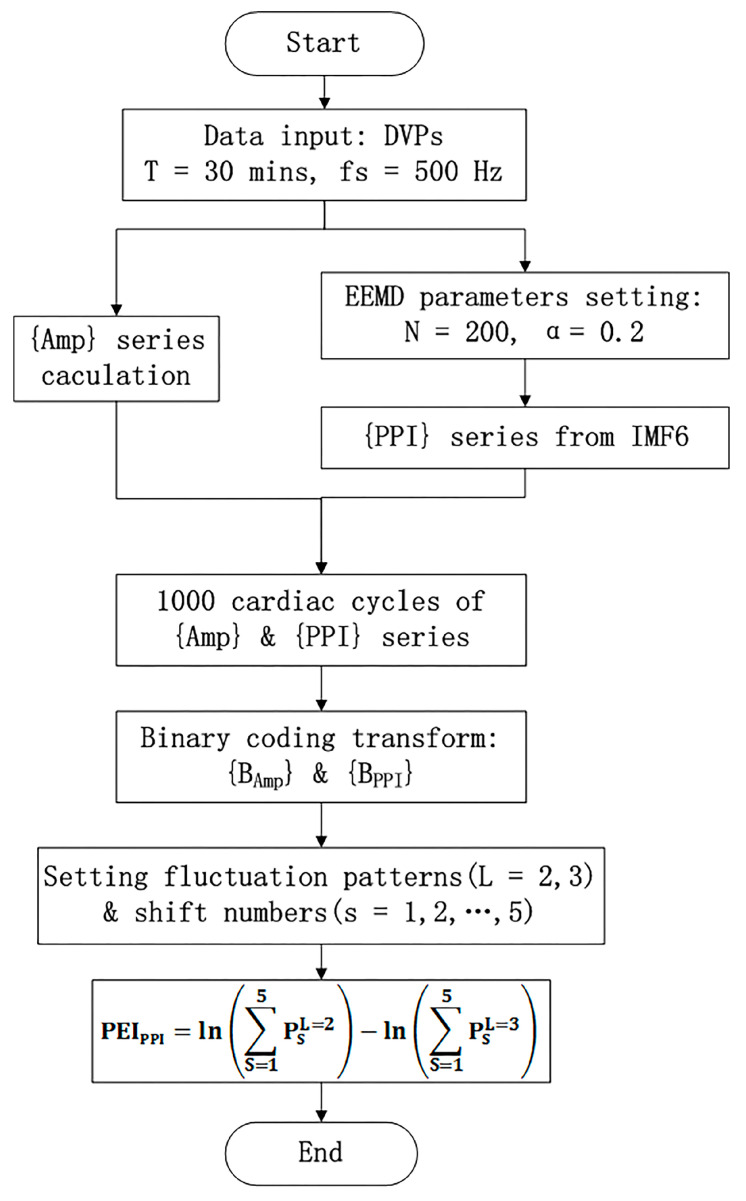
The modified percussion entropy index (PEI_PPI_) computation flow chart. Amp: DVP amplitudes of the dominant finger; PPI: peak-to-peak interval of IMF6. The standard deviation of the added noise was set as α = 0.2, and the trial number of the ensemble N = 200 for ensemble empirical mode decomposition (EEMD). After two synchronized series, {Amp} and {PPI} series were acquired. The computational length of the series {Amp} and {PPI} was set as 1000. Taking into account baroreflex sensitivity (BRS) regulation, the binary sequence transformations for {Amp} and {PPI} were conducted. Subsequently, the proposed PEI_PPI_ was computed as Equation (10).

- Fluctuation patterns with length of two
  Any two consecutive values [a_1_ a_2_] form one of the four fluctuation patterns, not six ordinal patterns, because only “ups and downs” are focused on BRS, as shown in Figure 3a. If one cardiac cycle delay exists between {Amp} and {PPI}, then {a_1_ a_2_ a_3_ ⋯⋯ a_n_} and {p_2_ p_3_ p_4_ ⋯⋯ p_n+1_} are used to match the obedience in fluctuation tendency with every two dimension counts, and then the percussion number (i.e., the numbers of matches) is acquired and divided by the total number of the two dimensions of fluctuation patterns. This is called percussion frequency with one cardiac cycle delay, and it is expressed by the following equation:
  (5) $$P_{s=1}^{L=2}=\frac{1}{n-2}\sum_{i=1}^{n-2}\mathrm{count}\left(i\right).$$
  If two cardiac cycle delays exist between {Amp} and {PPI}, then {a_1_ a_2_ a_3_ ⋯⋯ a_n_} and {p_3_ p_4_ p_5_ ⋯⋯ p_n+2_} are used for matching obedience in the fluctuation tendency with every two dimension counts, and then the percussion number (i.e., the number of matches) is acquired and divided by the total number of the two dimensions of fluctuation patterns. This is called percussion frequency with two cardiac cycle delays, expressed by the following equation:
  (6) $$P_{s=2}^{L=2}=\frac{1}{n-2}\sum_{i=1}^{n-2}\mathrm{count}\left(i\right).$$
  Thus, percussion frequency with five cardiac cycle delays between {Amp} and {PPI} is expressed as follows:
  (7) $$P_{s=5}^{L=2}=\frac{1}{n-2}\sum_{i=1}^{n-2}\mathrm{count}\left(i\right).$$
  Hence, the percussion entropy with length two of obedience in the fluctuation tendency can be defined as follows:
  (8) $$\varphi^{L=2}\left(n\right)=ln\left(\sum_{s=1}^{5}P_{s}^{L=2}\right),\;\;ln:\mathrm{natural}\;\mathrm{logarithmic}\;\mathrm{operation}.$$
  The higher the value in (8), the higher the BRS.
- Fluctuation patterns with length three
  Similarly, the percussion entropy with length three of obedience in the fluctuation tendency can be expressed as follows:
  (9) $$\varphi^{L=3}\left(n\right)=ln\left(\sum_{s=1}^{5}P_{s}^{L=3}\right),\;\;ln:\mathrm{natural}\;\mathrm{logarithmic}\;\mathrm{operation}.$$
  The higher the value in Equation (9), the lower the human biological complexity.
- PEI_PPI_ calculation
  (10) $${\mathrm{PEI}}_{\mathrm{PPI}}=\varphi^{L=2}\left(n\right)-\varphi^{L=3}\left(n\right).$$
  The calculation of the new percussion entropy index (PEI_PPI_) comprises the flow chart shown in Figure 4. Compared with PEI_PPI_, PPG and ECG signals were synchronized and sampled on the same system. The previous parameter (i.e., PEI_RRI_) was then computed from {Amp} and {RRI} for every subject. In addition, LHR_RRI_ and MEI_RRI_ were computed with only the RRI dataset used for comparison.

### 2.4. Statistical Analysis

All values in the Table 1 and Table 2 are expressed as the mean ± SD. The Statistical Package for the Social Sciences (SPSS, version 14.0 for Windows, SPSS Inc. Chicago, IL, USA), a powerful and user-friendly software package for statistical analysis of data, was adopted for all statistical analyses in the study. A one-sample Kolmogorov–Smirnov test in SPSS was adopted by testing the normality of the distribution, and the homoscedasticity of the data was verified subsequently. The comparisons of demographic, hemodynamic, anthropometric, and serum biochemical information of the test subjects were analyzed using a Student’s unpaired t test with Bonferroni correction, and the differences between categorical variables were assessed using a chi-square test. A Bland–Altman plot and Pearson’s correlation test with Bonferroni correction were performed for verification of the statistical agreement and judgment between PPI (IMF6) and PPI (DVP). The significance of differences in anthropometric, hemodynamic, and parameters (i.e., PEI_PPI_, PEI_RRI_, MEI_RRI_, and LHR_RRI_) among different groups were illustrated using independent sample *t*-tests with Bonferroni correction. The Pearson’s correlation test in SPSS was also adopted for the correlations verification between risk factors and the compared parameters. A corrected *p*-value with a test-specific significance level of 0.017 was regarded as statistically significant.

## 3. Results

### 3.1. Characteristics of the Age-Controlled Healthy and Diabetic Subjects

Table 1 shows the baseline characteristics of the three age-controlled groups. Compared with test subjects in the healthy group (i.e., group 1), those in the diabetes groups (i.e., group 2 and group 3) had higher weight, body mass index, triglyceride, fasting blood glucose, and glycosylated hemoglobin levels (*p* < 0.001). There were no notable differences between group 2 and group 3 in terms of demographic (i.e., age), anthropometric (i.e., body weight and waist circumference), hemodynamic (i.e., SBP, DBP, PP), and serum biochemical information (i.e., triglycerides, glycated hemoglobin, and fasting blood glucose) of the test subjects (*p* > 0.017).

### 3.2. Failure of DVPs to Detect Peak-to-Peak Intervals in an Aged Subject and a Diabetic Subject

**Figure 5:**
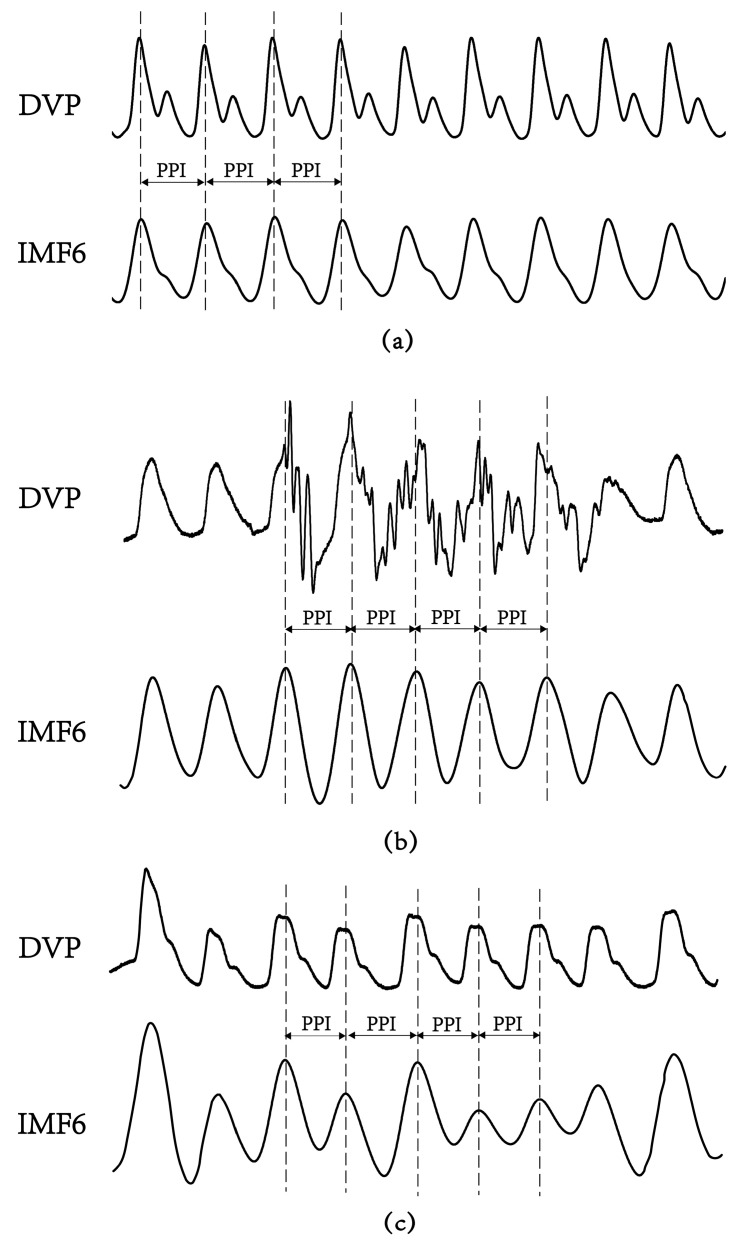
Digital volume pulse (DVP) signals and their corresponding decomposed 6th intrinsic mode function (IMF6) from one representative subject in each group showing: (**a**) subject A: healthy elderly subject in group 1 (age: 55 years, HbA1c: 5.5%, WC: 73 cm, BMI: 22.7); (**b**) subject B: diabetic patient without peripheral neuropathy in group 2 (age: 71 years, HbA1c: 8.2%, WC: 94 cm, BMI: 26.5); (**c**) subject C: type 2 diabetic patient with peripheral neuropathy within 5 years in group 3 (age: 62 years, HbA1c: 8.5%, WC: 98 cm, BMI: 28.6). The peaks of DVP and IMF6 were in phase for subject A, {PPI} series in (2) were the same from DVP or from IMF6. Nevertheless, it was difficult to calculate exact PPI from DVP for subject B and subject C. (**a**–**c**) For all IMF6 values, exact PPI could be calculated.

Figure 5 shows the failure of the digital volume pulse measured at the dominant fingertip in detecting the peak-to-peak interval (PPI) in an aged subject and a diabetic subject, as compared with the chaotic and subtle peaks of a middle-aged nondiabetic subject. The tangled results can be attributed to the interfering noises, including the signals of impaired peripheral circulation, respiration, involuntary vibrations, and whispering cough, and also mechanical signals such as those from the system of measurements. By implemented EEMD in the study, these noises were then removed to obtain refined DVP signals (i.e., IMF6) for exact PPI calculation.

### 3.3. Agreement Assessment between PPI (DVP) and PPI (IMF6) for the Three Groups

**Figure 6:**
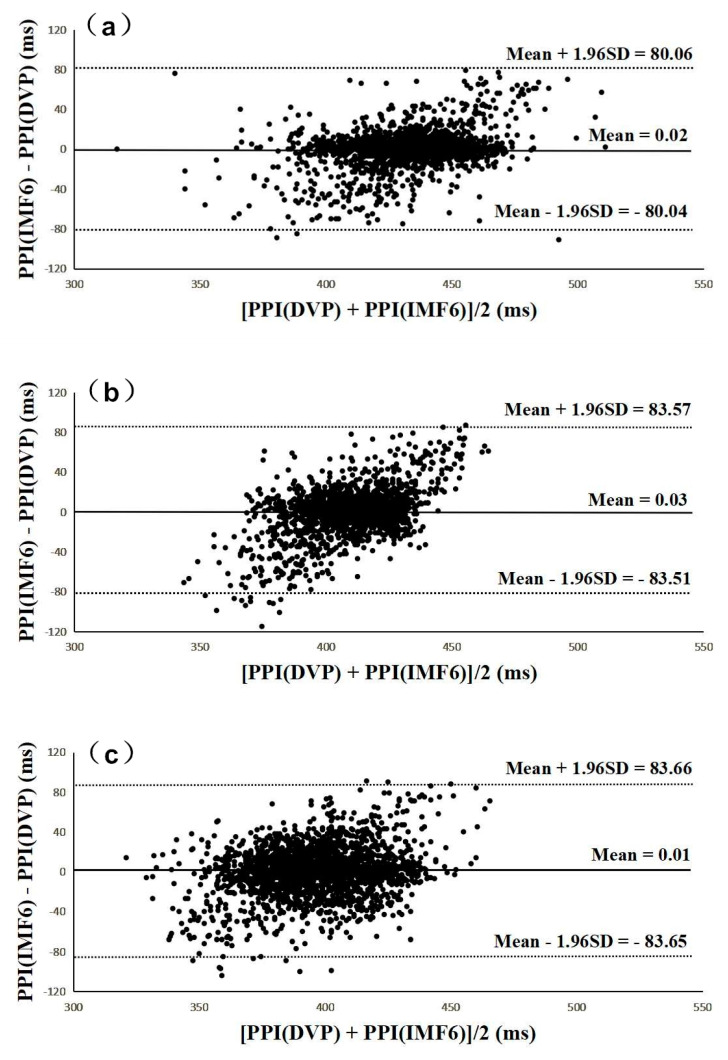
All Bland–Altman plots demonstrated that PPI (DVP) series have a good agreement with PPI (IMF6) series for (**a**) group 1, (**b**) group 2, and (**c**) group 3. Group 1: healthy aged subjects; group 2: diabetic subjects; group 3: diabetic peripheral neuropathy patients. The mean difference and the limits of agreement (mean ± 1.96SD) are also represented.

**Figure 7:**
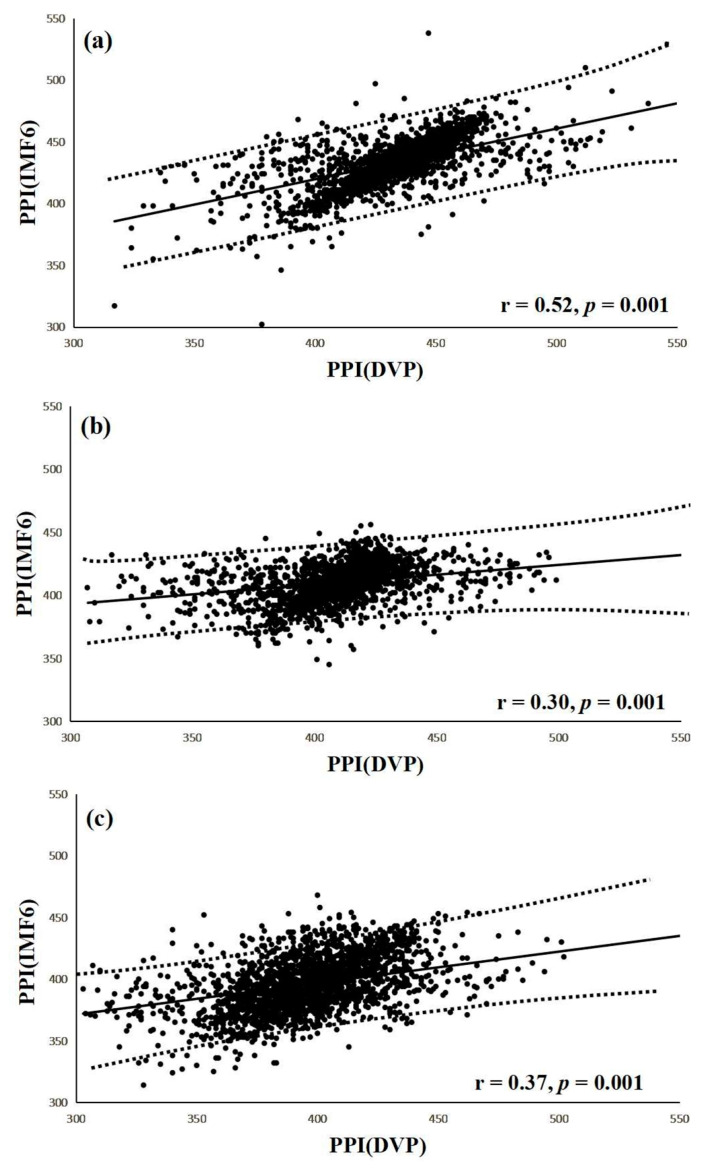
(**a**) Correlation between PPI (IMF6) and PPI (DVP) for test subjects in group 1 (r = 0.52, *p* = 0.001); (**b**) Correlation between PPI (IMF6) and PPI (DVP) for test subjects in group 2 (r = 0.30, *p* = 0.001); (**c**) Correlation between PPI (IMF6) and PPI (DVP) for test subjects in group 3 (r = 0.37, *p* = 0.001). Group 1: healthy aged subjects; group 2: diabetic patients without DPN; group 3: diabetic peripheral neuropathy patients within 5 years. The regression line describes the 95% confidence interval.

Figure 6 and Figure 7 were added to verify the hypothesis: the IMF6 signal can replace DVP for PPI determination. Figure 6 shows Bland–Altman plots of these two measurements (i.e., PPI (IMF6) and PPI (DVP)) for subjects from the three groups. The mean difference and the limits of agreement (mean ± 1.96SD) are also indicated in Figure 6. Good agreements were again shown between the two measurements for all test subjects.

Interestingly, a significant correlation was noted between PPI (IMF6) and PPI (DVP) in group 1 subjects (r = 0.52, *p* = 0.001) (Figure 7a). Similarly, notable correlations were also demonstrated in group 2 and group 3 (r = 0.30, r = 0.37, respectively, all *p* = 0.001) (Figure 7b,c). The regression line describes the 95% confidence interval in Figure 7.

### 3.4. Performance Compared among PEI_PPI_, PEI_RRI_, MEI_RRI_, and LHR_RRI_ to Differentiate Future Peripheral Neuropathy from Type 2 Diabetic Patients

The results of comparing the three previous parameters (i.e., LHR_RRI_, MEI_RRI_, and PEI_RRI_) with the proposed PEI_PPI_ for autonomic function and BRS evaluation in test subjects of the three groups are shown in Table 2. Although MEI_RRI_ was significantly higher for test subjects in group 1 than those in group 2 (p < 0.001), there was no notable difference between the test patients in groups 2 and 3. Relatively speaking, PEI_PPI_ and PEI_RRI_ successfully discriminated for the test subjects among the three groups with significant differences (p < 0.017) (Table 2).

### 3.5. Correlations of Risk factors with PEI_PPI_, PEI_RRI_, MEI_RRI_, and LHR_RRI_

The associations of the computational parameters (i.e., LHR_RRI_, MEI_RRI_, PEI_RRI_, and PEI_PPI_) with the anthropometric (i.e., body weight and waist circumference) and serum biochemical (i.e., triglycerides, fasting blood glucose, and glycated hemoglobin) factors of the test subjects in three groups were delivered and analyzed using the Pearson’s correlation test in SPSS (Table 3).

## 4. Discussion

Although a previous study [19] showed that PPG-based PPI could be reliably used for HRV computation, other authors [18,23,28] stated that PPI should be used carefully in overweight, elderly, or diabetic individuals. In contrast with the major problems focused at the beginning of this study are the chaotic and subtle peaks of DVP signals obtained from the fingertip (as shown in Figure 1 and Figure 5). EEMD is a nonlinear technique based on the orthogonal decomposition resulting from IMF6 being analyzed effectively in overweight, elderly, or diabetic individuals for exact PPI determination. In this way, the confusing noises were separated, including the signals of impaired peripheral circulation, respiration, involuntary vibrations, and whispering cough; as well as mechanical signals, such as those from the measuring system. Using EEMD in this study, these noises were separated to acquire refined DVP signals (i.e., IMF6) for exact PPI calculation on clinical applications (Figure 6 and Figure 7). 

Type 2 diabetes and related complications are related with the long-term damage and failure mechanisms of various organ systems [29]. The impact of lowering glucose on vascular complications and clinical outcomes in type 2 diabetes is still an open problem. While intensive glucose control in patients with type 2 diabetes has undoubted benefits for major microvascular endpoints [30,31,32,33], good glucose control improves microvascular disease, and should be achieved early and maintained over a very long period of time. Previous reviews [29,33] highlighted the need for program implementation for early screening, detection, and awareness to reduce the burden of managing complications. Diabetic peripheral neuropathy (DPN) is not only one of the most common chronic complications of diabetes, but also a leading cause for disability due to foot ulceration and amputation, fall-related injury, and gait disturbance [3]. Hence, this study addressed results from the indices LHR_RRI_, MEI_RRI_, PEI_RRI_, and PEI_PPI_, which were first computed for diabetic subjects with peripheral neuropathy within five years after PPG baseline measurement (i.e., group 3) for comparison with diabetic patients without peripheral neuropathy in the same period (i.e., group 2). However, the value of autonomic function indices, including MEI_RRI_, were significantly different in group 2 compared with group 1 subjects (*p* < 0.017). There were no notable differences between groups 3 and 2 (*p* > 0.017) (Table 2). On the other hand, both baroreflex sensitivity assessment indices (i.e., PEI_RRI_ and PEI_PPI_) showed highly significant differences among the three groups (*p* < 0.017) (Table 2). Significantly smaller values of PEI_PPI_ were noted for group 3 compared to the other two groups (e.g., group 1 versus group 2 versus group 3: 0.69 ± 0.03 versus 0.66 ± 0.04 versus 0.63 ± 0.06, respectively), which is consistent with the same finding that diabetic peripheral neuropathy was found to be a more important determining factor of spontaneous BRS assessment than elasticity of carotid arteries in type 2 diabetics [34]. 

Although PEI_RRI_ has recently been reported to assess the complexity of BRS [16,17], the significance of smaller PEI_RRI_ values concerning the identification of subjects with type 2 diabetes who are more prone to develop DPN is unknown. In addition, there were no notable differences between the test diabetic patients in groups 2 and 3 with anthropometric, demographic, hemodynamic, and serum biochemical parameters (*p* > 0.017) (Table 1). That is to say, it would be difficult to predict how many and who will develop peripheral neuropathy in advance. Finally, 24 diabetic patients in the study were identified with peripheral neuropathy (i.e., group 3): 10 subjects had good blood glucose control (i.e., 6.5% ≦ HbA1c < 8%), and the other 14 subjects had poor blood glucose control (i.e., HbA1c ≧ 8%). Thus, nearly 42% (10 out of 24) of DPN patients originally had good blood glucose control but developed further peripheral neuropathy within five years after PPG baseline measurement. The impact of poor glycemic control and sedentary status are well-known risk factors of DPN [35,36]. These results are consistent, finding that the associations of the PEI_RRI_ and PEI_PPI_ indices with the anthropometric (i.e., body weight and waist circumference) and serum biochemical (i.e., triglycerides, glycated hemoglobin, and fasting blood glucose) parameters of all test subjects were noted (Table 3). Significantly, peripheral neuropathy dysfunction has been associated with the development of macrovascular diseases, such as ischemic stroke, peripheral vascular disease, and acute myocardial infarction in diabetes [37,38,39]. 

This study has some limitations. First, the number of test subjects enrolled was relatively low. Second, this was an outpatient clinical study, and details on dietary information and medical management of diabetes may not be integrated. Third, real-time processing and stream processing were not possible for PEI_PPI_ computation because EEMD required lots of operations. Immediate PEI_PPI_ information available to the examinees, therefore, could not be provided for the test subjects. This limitation may hopefully be overcome by the application of a real time Labview-based package in the future.

## 5. Conclusions

The results of this study not only indicate that PPG-derived digital volume pulse signals capable of being de-noised by ensemble empirical mode decomposition may be an important contributor to peak-to-peak interval detection success for aged overweight subjects and diabetic patients, but also recommend the possibility of clinical application of a modified percussion entropy index (i.e., PEI_PPI_) for DVP signals used only as a simple and noninvasive prognostic indicator for diabetic patients with peripheral neuropathy dysfunction. This manuscript reported a study to investigate the application of a modified percussion entropy index in accessing the complexity of baroreflex sensitivity for diabetic peripheral neuropathy prognosis. On the other hand, there are no obvious symptoms at their early stages. Early detection of the signs of diabetic peripheral neuropathy through signal analysis methods, therefore, is of utmost importance in the field of preventive medicine that requires collaborative efforts of clinicians and medical technologists.

## Figures and Tables

**Table 1:** Characteristics of the study population.

Parameters	Group 1	Group 2	Group 3
	Number: 34	Number: 42	Number: 24
	Female/Male	Female/Male	Female/Male
	(15/19)	(14/28)	(10/14)
Age, year	59.78 ± 9.74	65.90 ± 11.10	62.26 ± 8.67
Body height, cm Body weight, kg WC, cm BMI, kg/m^2^	160.95 ± 7.16 61.51 ± 10.02 82.56 ± 10.57 23.59 ± 3056	159.58 ± 7.97 68.74 ± 10.31 ** 93.85 ± 8.99 27.02 ± 3.93 **	164.35 ± 9.67 71.98 ± 7.54 95.44 ± 6.88 26.85 ± 3.94
SBP, mmHg DBP, mmHg PP, mmHg HDL, mg/dL LDL, mg/dL Cholesterol, mg/dL	123.88 ± 18.69 75.01 ± 8.99 48.88 ± 14.41 51.76 ± 21.05 113.23 ± 30.25 190.08 ± 48.99	128.80 ± 16.61 75.66 ± 10.35 51.88 ± 16.05 45.14 ± 17.82 124.11 ± 47.08 174.53 ± 50.79	125.09 ± 31.64 72.01 ± 18.04 53.09 ± 18.76 39.32 ± 6.18 106.23 ± 25.08 179.78 ± 31.38
TG, mg/dL	94.81 ± 36.16	148.95 ± 67.84 **	164.18 ± 68.05
HbA1c, %	5.92 ± 0.32	7.99 ± 1.68 **	8.48 ± 1.58
FBS, mg/dL	100.42 ± 26.80	150.73 ± 48.52 **	162.69 ± 58.24

All values are expressed as mean ± SD. Group 1: healthy aged subjects; group 2: diabetic patients without peripheral neuropathy; group 3: type 2 diabetic patients with peripheral neuropathy within 5 years. BMI: body mass index; WC: waist circumference; SBP: systolic blood pressure; DBP: diastolic blood pressure; PP: pulse pressure; LDL low-density lipoprotein cholesterol; HDL: high-density lipoprotein cholesterol; TG: triglyceride; HbA1c: glycosylated hemoglobin; FBS: fasting blood glucose. Note: ** *p* < 0.001 group 1 versus group 2, a *p*-value < 0.017 was noted as statistically significant.

**Table 2:** Performance comparison in BRS and autonomic function assessment among the three groups of test subjects.

Parameters	Group 1 (n = 34)	Group 2 (n = 42)	Group 3 (n = 24)
LHR_RRI_	1.59 ± 1.03	2.09 ± 2.09	2.25 ± 2.38
MEI_RRI_	0.49 ± 0.16	0.36 ± 0.20 **	0.37 ± 0.19
PEI_RRI_	0.73 ± 0.47	0.60 ± 0.11 **	0.56 ± 0.10 ^†^
PEI_PPI_	0.69 ± 0.03	0.66 ± 0.04 **	0.63 ± 0.06 ^†^

All values are expressed as the mean ± SD. Group 1: healthy aged subjects; group 2: diabetic patients without peripheral neuropathy; group 3: diabetic peripheral neuropathy patients. PEI_PPI_: percussion entropy index using synchronized {PPI} and {Amp} signals; PEI_RRI_: percussion entropy index using synchronized RRI and Amp signals; MEI_RRI_: mean value of sample entropy on a scale from 1 to 5 using the RRI dataset only; and LHR_RRI_: low-/high-frequency power ratio using the RRI dataset only. ** *p* < 0.001 group 1 versus group 2, ^†^ *p* < 0.017 group 2 versus group 3, a *p*-value less than 0.017 was noted as statistically significant.

**Table 3:** Associations of anthropometric and serum biochemical risk factors with parameters in all test subjects.

	PEI_PPI_		PEI_RRI_		MEI_RRI_		LHR_RRI_	
	r	*p*	r	*p*	r	*p*	r	*p*
BW, kg	−0.20	0.04	−0.19	0.06	−0.07	0.53	−0.07	0.53
WC, cm	−0.20	0.05	−0.31	0.00	−0.02	0.85	−0.01	0.95
TG, mg/dL	−0.27	0.01	−0.33	0.00	−0.09	0.39	0.05	0.64
HbA1c, %	−0.38	0.00	−0.43	0.00	−0.26	0.01	−0.14	0.17
FBS, mg/dL	−0.23	0.03	−0.40	0.00	−0.28	0.01	−0.05	0.63

PEI_PPI_: percussion entropy index using synchronized {PPI} and {Amp} signals; PEI_RRI_: percussion entropy index using synchronized RRI and {Amp} signals; MEI_RRI_: mean value of sample entropy on a scale from 1 to 5 using the RRI dataset only; and LHR_RRI_: low-/high-frequency power ratio using the RRI dataset only. BW: body weight; WC: waist circumference; TG: triglyceride; HbA1c: glycosylated hemoglobin; FBS: fasting blood glucose. A *p*-value less than 0.05 was noted as statistically significant.

## Abbreviations

BMI	Body Mass Index
BRS	Baroreflex Sensitivity
DBP	Diastolic Blood Pressure
DVP	Digital Volume Pulse
DPN	Diabetic Peripheral Neuropathy
ECG	Electrocardiography
EEMD	Ensemble Empirical Mode Decomposition
FBS	Fasting Blood sugar
HbA1c	Glycosylated hemoglobin
HDL	High-Density Lipoprotein cholesterol
HFP	High Frequency Power
HRV	Heart Rate Variability
IMF6	The 6th decomposed Intrinsic Mode Function
LDL	Low-Density Lipoprotein cholesterol
LFP	Low Frequency Power
LHR_RRI_	Low-/High-frequency power Ratio (LFP/HFP, LHR) using the RRI dataset
MEI_RRI_	Multiscale Entropy Index using the RRI dataset only
PC	Personal Computer
PEI_PPI_	Percussion Entropy Index using synchronized {PPI} and {Amp} signals
PEI_RRI_	Percussion Entropy Index using synchronized {RRI} and {Amp} signals
PP	Pulse Pressure
PPG	Photoplethysmography
PPI	Peak-to-Peak Interval
RRI	R-R Interval of ECG
SBP	Systolic Blood Pressure
SPSS	Statistical Package for the Social Sciences
TG	Triglyceride
WC	Waist Circumference
